# Pathophysiological Changes of Physical Barriers of Peripheral Nerves After Injury

**DOI:** 10.3389/fnins.2018.00597

**Published:** 2018-08-23

**Authors:** Qianyan Liu, Xinghui Wang, Sheng Yi

**Affiliations:** Key Laboratory of Neuroregeneration of Jiangsu and Ministry of Education, Co-innovation Center of Neuroregeneration, Nantong University, Nantong, China

**Keywords:** peripheral nerve, physical barrier, blood-nerve barrier, cellular junction, peripheral nerve injury

## Abstract

Peripheral nerves are composed of complex layered anatomical structures, including epineurium, perineurium, and endoneurium. Perineurium and endoneurium contain many physical barriers, including the blood-nerve barrier at endoneurial vessels and the perineurial barrier. These physical barriers help to eliminate flux penetration and thus contribute to the establishment of a stable microenvironment. In the current review, we introduce the anatomical compartments and physical barriers of peripheral nerves and then describe the cellular and molecular basis of peripheral physical barriers. We also specifically explore peripheral nerve injury-induced changes of peripheral physical barriers, including elevated endoneurial fluid pressure, increased leakage of tracer, decreased barrier-type endothelial cell ratio, and altered distributions and expressions of cellular junctional proteins. The understanding of the pathophysiological changes of physical barriers following peripheral nerve injury may provide a clue for the treatment of peripheral nerve injury.

## Introduction

Peripheral nerves, different from central nerves are protected by vertebral column, skull, or the blood-brain barrier, are delicate and easily damaged nerve tissues. However, in the peripheral nervous system, there still exists the blood-nerve barrier. The blood-nerve barrier is composed of physical barrier at endoneurial vessels within the nerve fascicle and the perineurial barrier (Weerasuriya and Mizisin, 2011). These physical barriers protect peripheral nerves from external influencing factors, help to maintain the homeostasis of the microenvironment of peripheral nerves, and thus play important physiological roles in the peripheral nervous system (Olsson, 1990; Weerasuriya and Mizisin, 2011).

These physical barriers in the peripheral nervous system may be altered and disrupted under various pathological conditions, especially many immune mediated neuropathies such as Guillain–Barré syndrome (GBS), chronic inflammatory demyelinating polyradiculoneuropathy (CIDP), multifocal motor neuropathy (MMN), and polyneuropathy, organomegaly, endocrinopathy, monoclonal protein, and skin changes (POEMS) (Kanda, 2013; Reinhold and Rittner, 2017; Skaper, 2017). Besides peripheral neuropathies, trauma to the peripheral nervous system can also lead to pathophysiological changes of these physical barriers (Mizisin and Weerasuriya, 2011; Reinhold and Rittner, 2017). In the current review, we introduce anatomic components of the peripheral nervous system, describe cellular and molecular elements of physical barriers of peripheral nerves, and specifically discuss pathophysiological changes of these physical barriers following peripheral nerve injury.

## Physical Barriers in Peripheral Nerves

### Anatomical Structures of Peripheral Nerves

**FIGURE 1:**
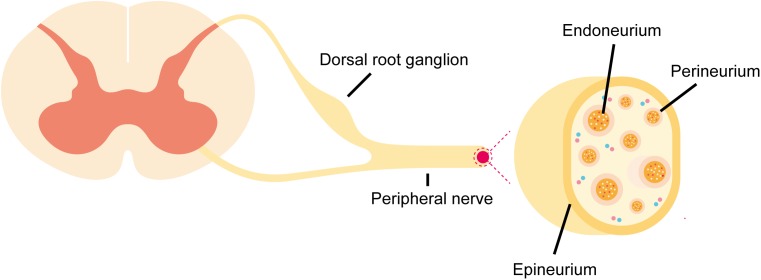
Schematic representation of anatomical structures of peripheral nerves. Peripheral nerve stump is composed of epineurium, perineurium, and endoneurium. The blood-nerve barrier is formed by physical barrier at endoneurial vessels and the perineurial barrier.

Peripheral nerves are mainly composed of somas embedded in the spinal cord, brain stem, dorsal root ganglia, sympathetic ganglia, or parasympathetic ganglia and extended axons that signal with target organs (Romero-Ortega, 2015). Axons are grouped into nerve fascicles or nerve bundles. Anatomically, peripheral nerves contain three layers of protective connective sheath: epineurium, perineurium, and endoneurium (**Figure 1**; Osawa et al., 1999; Verheijen et al., 2003; Siemionow and Brzezicki, 2009). Epineurium is the outermost layer of dense tissue component that encloses the entire peripheral nerve. Epineurium encircles numerous nerve fascicles, blood vessels, lymphocytes, and fibroblasts. Perineurium is the middle layer that wraps around each nerve fascicle. Perineurium is not a single layer tissue but a connective tissue of about 7–8 concentric layers. Perineurial cells are also called myoepithelial cells because they contain certain epithelioid and myofibroblastoid properties, including the presence of cellular junctions and the ability of contraction. Therefore, perineurium is elastic and resistant to certain mechanical damage. Endoneurium, so called endoneurial tube, endoneurial channel, or Henle’s sheath, is the innermost layer. Endoneurium surrounds a cluster of small unmyelinated nerve fibers or more commonly, the myelin sheath of each myelinated nerve fiber (Ubogu, 2013). Endoneurially, myelinating Schwann cells wrap around axons in multiple layers, form the myelin sheath, and thus constitute a protective and isolated structure. Endoneurial vessels form a fine capillary network and provide oxygenic and nutritional supply (Grinsell and Keating, 2014). Endothelial cells in the endoneurial vessels also form a physical barrier called blood-nerve barrier that protects the homeostasis of the endoneurial space.

### Blood-Nerve Barrier at Endoneurial Vessels

Endoneurial vessels are composed of a network of arterioles, non-fenestrated capillaries, and venules (Weerasuriya and Mizisin, 2011). This vascular network consists of specialized endothelial cells that form tight inter-endothelial junctions and occasional open inter-endothelial gaps. Different from endothelial cells in epineurium or perineurium that possess fenestration, endothelial cells in endoneurial vessels are normally non-fenestrated cells with dense and rich intercellular tight junction structures (Kanda, 2013; Ubogu, 2013). Besides endothelial cells in endoneurial vessels, endothelial pericytes also express tight junction proteins and barrier-related transporters, possess high trans-pericyte electrical resistance values, and exhibit barrier-forming properties (Shimizu et al., 2008).

However, due to the lack of astrocytes, the blood-nerve barrier showed significant differences when comparing with the blood-brain barrier. The blood-nerve barrier is considered to be relatively much leakier than the blood-brain barrier (Kanda, 2013). To quantify the permeability of the blood-nerve barrier, blood-nerve barrier endoneurial endothelial cells were isolated, purified, and cultured (Yosef et al., 2010; Abe et al., 2012; Yosef and Ubogu, 2013). The hydraulic conductivity of cultured primary and immortalized human endoneurial endothelial cells was 10 times higher than the conductivity of immortalized human brain microvascular endothelial cells, a commonly used blood-brain barrier cell line (Helton et al., 2017). However, the blood-nerve barrier is still a tight physical barrier since it was demonstrated that the permeabilities of some impermeable proteins, such as insulin, albumin, transferrin, and immunoglobulin G, as well as neurotrophic factors, such as nerve growth factor, ciliary neurotrophic factor, neurotrophic-3, and brain-derived neurotrophic factor at the blood-nerve barrier and the blood-brain barrier were similar with each other (Poduslo et al., 1994; Poduslo and Curran, 1996). Measurement of the transendothelial electrical resistance also showed that confluent blood-nerve barrier endoneurial endothelial cells reached a maximum transendothelial electrical resistance of 160–180 Ω cm^2^, a comparative value as that of blood-brain barrier endothelial cells (Yosef et al., 2010; Yosef and Ubogu, 2013).

Blood-nerve barrier endoneurial endothelial cells also express a variety of transporters on both the luminal and the abluminal sides. The expressions of γ-glutamyl transpeptidase, the Na^+^-independent L-type amino acid transporter-1, creatine transporter, monocarboxylate transporter-1, glucose transporter-1, alkaline phosphatases, and P-glycocoprotein in cultured blood-nerve barrier endothelial cells was detected by PCR, Western blot, or immunodetection (Yosef et al., 2010; Ubogu, 2013; Yosef and Ubogu, 2013). RNA sequencing further demonstrated the presence of the transcripts of numerous transporters, including members of the solute carrier transporter family, ATP-binding cassette transporter family, cation channels, anion channels, zinc transporters, solute carrier organic anion transporters, and aquaporins, in cultured primary human endoneurial endothelial cells and laser-capture microdissected human sural endoneurial microvessels (Palladino et al., 2017). These findings suggested that the blood-nerve barrier is not only a physical fence but also an active and competent interface (Mizisin and Weerasuriya, 2011; Weerasuriya and Mizisin, 2011; Kanda, 2013).

The permeability of physical barriers is also affected by ion distributions. Examinations of the distributions of anionic microdomains in rat sciatic nerve vascular endothelium with cationic tracers showed there existed high densities of anions in diaphragms of caveolae and transendothelial channels, and luminal endothelial processes, moderate densities of anions in the basal laminae of endothelial cells, pericytes, and luminal membranes, low densities of anions in abluminal membranes, and no anions in the inter-endothelial tight junctions (Bush and Allt, 1990). Further investigation of the nature of endothelial anionic sites in peripheral nerves showed that sialic acid-containing glycoproteins were principally responsible for these anionic sites (Lawrenson et al., 1994).

### Perineurial Barrier

Perineurial cells are central nervous system-derived cells that obtain glial cell properties (Kucenas et al., 2008; Morris et al., 2017). Besides endoneurial endothelium, perineurium is generally considered to be another critical structure that composes the blood-nerve barrier (Reinhold and Rittner, 2017). Light and electron microscopy studies revealed that there existed apparent and abundant tight junction strands between neighboring perineurial cells (Thomas, 1963; Gamble and Eames, 1964; Reale et al., 1975; Beamish et al., 1991). Tight junction proteins were also detected in human perineurium (Pummi et al., 2004). These tight junction structures help to form a physical perineurial barrier that can protect internal axons from ions, toxins, and other external materials (Kucenas, 2015). Morphological observations provide further evidence of the barrier function of perineurial cells. For example, few penetration of the electron-dense tracer was observed after the *in vivo* microinjection of lanthanum nitrate into rat sural and tibial nerves (Ghabriel et al., 1989). No penetration of lanthanum was observed in opossum sciatic nerve perineurium not only in normal conditions but also following the treatment of detergent bile salt sodium deoxycholate (Todd et al., 2000). Meanwhile, it was also identified that perineurial cells contained pinocytotic vesicles and could mediate active transcytotic transport (Oldfors, 1981; Peltonen et al., 2013).

## Molecular Components of Cellular Junctions

The blood-nerve barrier at endoneurial vessels and the perineurial barrier jointly restrict the exchanges of materials between the endoneurial space and the surrounding extracellular space and thus contribute to the relative stability of neural microenvironment. The molecular bases of these physical barriers are cellular junctional proteins, especially tight junction proteins and adherens junction proteins. These cellular junctional proteins in peripheral physical barriers are briefly introduced here.

### Tight Junction Proteins

Tight junctions, also named occluding junctions or zonulae occludentes, are multimolecular complexes that seal the intercellular space and effectively prevent the passage of ions and molecules. Tight junctions are mainly composed of claudins and occludin. At least 24 isoforms of claudins have been identified in mammals. Many isoforms, including claudin-1, claudin-4, claudin-5, claudin-11, claudin-12, and claudin-19 were found in cultured human endoneurial endothelial cells (Yosef et al., 2010; Abe et al., 2012; Ubogu, 2013; Palladino et al., 2017). Claudin-1 and claudin-3 but not claudin-2 and claudin-4 were found in human perineurium. The expression of occludin was also determined in the physical barriers of peripheral nerves (Pummi et al., 2004; Ubogu, 2013). Meanwhile, the presences of tight junction-associated proteins zona occludens (ZO-1 and ZO-2) were identified as well (Pummi et al., 2004; Abe et al., 2012). Those tight junction-associated proteins serve as scaffolds and link claudins or occludin to the actin cytoskeleton.

### Adherens Junction Proteins

Adherens junctions, also named intermediate junctions or belt desmosome, are normally located in a more basal position than tight junctions. They are also important cellular junctions that mediate cell-cell adhesion. Adherens junctions are mainly composed of the cadherin-catenin complex. Cadherin, especially classical type epithelial-cadherin (E-cadherin), binds to β-catenin. β-catenin further binds to α-catenin and anchors to the actin cytoskeleton. PCR and Western blot outcomes showed that vascular endothelial-cadherin (VE-cadherin) and β-catenin were expressed in endoneurial endothelial junctions (Yosef and Ubogu, 2012). Moreover, a high-throughput transcriptome analysis of human endoneurial endothelial cells revealed the presences of other isoforms of cadherin and catenin (N-cadherin, osteoblasts-cadherin, heart-cadherin, myotubule-cadherin, kidney cadherin, cadherin 24, α1 catenin, δ1 catenin, and γ catenin) and other adherens junction proteins (protocadherins and nectin cell adhesion molecules) (Palladino et al., 2017).

These cellular junction proteins jointly seal the intercellular space between adjacent cells, regulate the paracellular movement, and contribute to a stable microenvironment under physiological conditions. However, under pathological conditions such as peripheral nerve injury, the localizations and/or expressions of these cellular junction proteins may be altered and physical barriers of peripheral nerves may be disrupted.

## Barrier Alternations Following Peripheral Nerve Injury

Peripheral nerve injury is a common clinical neurological disorder that affects about 20 million people and costs approximately 150 billion dollars annually in the United States (Lundborg and Richard, 2003; Taylor et al., 2008; Grinsell and Keating, 2014). Peripheral nerve injury can be induced by various causes, such as crush, stretch, sharp instrument injury, firearm injury, heat or cold-induced damage, and medical disorder (Campbell, 2008). Histological and morphological studies demonstrated that axons and surrounding myelin sheaths are disrupted and collapsed after peripheral nerve injury. Axons in the distal peripheral nerve stumps disconnect from neuron cell bodies and undergo Wallerian degeneration (Coleman, 2005; Geuna et al., 2009). Axons in the proximal peripheral nerve stumps undergo chromatolysis and degenerate back to the first node of Ranvier (Menorca et al., 2013; Grinsell and Keating, 2014).

The integrity of physical barriers in the peripheral nervous system is also affected by nerve injury. However, our understanding about the alternations of physical barriers and the internal microenvironment of peripheral nerves following peripheral nerve injury is very limited. Several parameters, such as values of endoneurial fluid pressure, the leakage of tracers, and the expressions of specific cell markers, have been used to determine the changes of physical barriers following nerve injury. Molecular changes of cellular junctional proteins are also detected.

### Increased Endoneurial Fluid Pressure

The pressure of endoneurial fluid is often measured to determine changes of fluid and/or electrolyte compositions in the endoneurial interstitium and to reflect alternations of physical barriers in the peripheral nervous system (Myers et al., 1983). For instance, the dynamic changes of endoneurial fluid pressure from day 0 to day 28 following rat sciatic nerve crush injury were recorded *in vivo* by inserting glass micropipettes directly into distal sciatic nerves. Endoneurial fluid pressure was significantly elevated within 90 min after sciatic nerve crush, reached to a 5-fold peak level at day 10, kept at a high level until day 16, and then declined to the basal level (Powell et al., 1979). The increase in endoneurial fluid pressure suggested that peripheral nerve injury would change the permeability across physical barriers in peripheral nerves and alter the local microenvironment (Low and Dyck, 1977; Powell et al., 1979). Since there is no lymphatic system in the endoneurial space, elevated permeability and imbalanced pressure can easily induce edema (Weerasuriya and Mizisin, 2011; Lim et al., 2014). Endoneurial edematous swelling was observed in sciatic nerve crush injury model as well as in many other mechanical peripheral nerve injury models, such as long-time placement of constrictive ligatures around rat common sciatic nerves (Bennett and Xie, 1988), ligation of half of rat sciatic nerves high in the thigh (Seltzer et al., 1990), compression in spinal nerve roots of the pig cauda equina (Olmarker et al., 1989), and the application of fixed-diameter polyethylene cuffs to rat sciatic or sural nerves (Mosconi and Kruger, 1996). Increased endoneurial fluid pressure was also detected in peripheral nerves with laser injury. Laser-produced rat sciatic nerve lesion caused Wallerian degeneration, induced perivascular and subperineurial edema in damaged areas and the entire fascicle, and led to elevated endoneurial fluid pressure at 2 and 6 days after sciatic nerve injury (Myers et al., 1985). Light microscopy observations also showed that laser-induced sciatic nerve injury would cause endoneurial edema at the irradiated site of rat sciatic nerves (Chiang et al., 2005). Besides heat-induced peripheral nerve injury, cold-induced injury changed endoneurial fluid pressure as well. Rat proximal sciatic nerves were exposed, frozen with specially designed probe to form an ice ball at -60°C for 60 s, and subjected to endoneurial fluid pressure measurement. After cryogenic injury, endoneurial fluid pressure elevated to about 11.5 folds within 90 min and did not return to the normal level until 32 days. Obvious separation of myelinated nerve fibers as well as fluid accumulation in perivascular and subperineurial space was also visualized by light microscopic examination, indicating that cryogenic injury would induce severe endoneurial edema (Myers et al., 1981).

### Increased Tracer Leakage

Tracer detection is also commonly used to determine the permeability of physical barriers in the peripheral nervous system. Sciatic nerve crush at different degrees (15 s single crush, 15 s double crush, 30 s single crush, 30 s double crush, 60 s single crush with a No. 5 jeweler’s forceps, and 30 s single crush with a serrated hemostat) all resulted in the leakage of Evans blue albumin complex from the blood vessels and through the perineurium into endoneurial space in rats at 2 days after nerve injury (Bridge et al., 1994). Sciatic nerve crush by ligation for 24 h induced the leakage of fluorescent Evans blue albumin into the connective tissue in the endoneurium from 1 to 5 days after rat nerve injury (Hirakawa et al., 2003). Partial sciatic nerve ligation led to the outflow of small-molecular fluorescent dye sodium fluorescein and the extravasation of plasma clotting factor fibrinogen at the injury and distal sites in mice from 3 to 28 days after nerve injury (Lim et al., 2014). Sciatic nerve transection in frogs induced increased vascular permeabilities to ^14^C-sucrose and horseradish peroxidase reaction product as well as enlarged vascular space (Latker et al., 1991). Leakages of tracers and fluorescent dyes were also detected in peripheral nerve injury-induced by other reasons except for trauma. For example, after cryogenic injury, horseradish peroxidase enzyme substrate complex was observed in both endoneurium and vessel lumens in rats injected with horseradish peroxidase into the jugular vein (Myers et al., 1981). Treating rat sciatic nerves with low temperatures (3–5°C) for 2 h would lead to non-freezing cold injury and result in the leakage of fluid tracer Evans blue in endoneurium, breakdown of the blood-nerve barrier, and severe edema in endoneurium (Li et al., 2015). Notably, it was demonstrated that the opening of tight junctions occurred at a later time point than the leakage of Evans blue (3 days after non-freezing cold injury versus within 1 day after injury). It suggested that at the acute phase after cold injury, the breakdown of the blood-nerve barrier might due to other factors besides the changes of tight junctions (Li et al., 2015). Similar as rats underwent crush and transection injuries, rats received intraneurial toxic ricin injection displayed extravasated fluorescein isothiocyanate labeled dextran (FITC-dextran) in the endoneurial and epineurial spaces after an intravascular injection of a 4,000 molecular weight FITC-dextran (Bouldin et al., 1991). Likewise, extravasation of fluorescent dextran was observed in endoneurium and epineurium of proximal sciatic nerve in rat intoxicated with tellurium (Bouldin et al., 1991).

### Decreased Ratio of Barrier-Type Endothelial Cells

The integrity of the blood-nerve barrier was also determined by immunohistochemical staining with endothelial cell markers. Omura et al. (2004) determined the numbers of barrier-type endothelial cells and total endothelial cells by cell markers anti-endothelial barrier antigen (anti-EBA) and anti-rat endothelial cell antigen-1 (anti-RECA-1), respectively. They further calculated the ratio of EBA positive cells to RECA-1 positive cells to assess the integrity of the blood-nerve barrier. Quantitative outcomes showed that the ratio of EBA/RECA-1 was decreased to about 1/3 of the normal value in the segment 5 mm proximal and the entire distal stump at 3 days after rat sciatic nerve crush, reached the valley value at 7 days, and then gradually covered (Omura et al., 2004).

### Disrupted Cationic Distribution

Moreover, the distributions of ions on the endothelial plasma membrane were found to be altered following peripheral nerve injury. Luminal labeling of unfenestrated epineurial and endoneurial vessels with cationic tracer cationic ferritin and colloidal gold showed that sciatic nerve crush would induce transient anionic fenestrations in a minority of mouse endoneurial vessels at 4 days after nerve crush. And the distributions of anions recovered to the normal state after 14 days (Bush et al., 1993).

### Altered Expressions and Localizations of Cellular Junctional Proteins

Molecular changes of cellular junctional proteins were also determined after injury to the peripheral nervous system. Immunohistochemical, morphometrical, and Westerns blot examinations showed that the expressions of gap junction proteins connexin 26, connexin 32, and connexin 43 were changed after peripheral nerve injury (Chandross et al., 1996; Chandross, 1998; Nagaoka et al., 1999). Outcomes from immunoconfocal microscopy and morphometry showed that intercellular junctional proteins claudin-1, claudin-5, occludin, VE-cadherin, and connexin 43 were not observed from perineurium and endoneurium at 1 day after ligation induced rat sciatic nerve crush injury. The localizations of claudin-1, claudin-5, occludin, and VE-cadherin were recovered at 2 days after injury while the localization of connexin 43 was recovered at 5 days (Hirakawa et al., 2003). Tight junction proteins occludin and ZO-1 as well as gap junction protein connexin 43 were not detectable at 3 days after perineurial window, a surgery method that caused limited peripheral nerve injury. Tight junction proteins occludin and ZO-1 were recovered and re-observed at 7 days after injury while gap junction protein connexin 43 were re-observed after 21 days (Ohta et al., 2005). Gene and protein expressions of claudin-5 and occludin were found to be decreased starting from 3 h after chronic constriction injury of rat sciatic nerves (Moreau et al., 2016). With the development of high-throughput screening, the dynamic changes of the expressions of cellular junctional proteins following peripheral nerve injury were further discovered. For instance, microarray analysis of rat distal nerve stumps showed that claudin-14 and claudin-15 were differentially expressed after sciatic nerve transection and were hub genes of the single-flow of early Wallerian degeneration (Li et al., 2013; Gong et al., 2014). Bioinformatic analysis also suggested that canonical signaling pathway tight junction was significantly involved in distal nerve stump after rat sciatic nerve transection injury (Cheng et al., 2017; Yi et al., 2017).

## Conclusion and Perspectives

Similar as brain and spinal cord that are protected by the blood-brain barrier and the blood-spinal cord barrier, respectively, peripheral nerves are also protected by protective physical barrier structures. The blood-nerve barrier helps to separate the inside of the peripheral nerve isolated from outside factors such as infections and toxins and thus builds a relatively stable internal microenvironment. Physical barriers in the peripheral nervous system are often disrupted under pathological conditions, especially immune mediated neuropathies. Although not well studied to date, physical barriers in the peripheral nervous system are also influenced and disrupted by peripheral nerve injury.

In the current review, anatomical structures and molecular components of physical barriers in the peripheral nervous system are described. Alternations in these physical barriers induced by peripheral nerve injury are then introduced. However, the roles of peripheral physical barriers in peripheral nerve repair and regeneration remain largely undiscovered. It was demonstrated that after zebrafish peripheral nerve injury, perineurial cells would extend to injury sites, clear cellular debris, and fill injury gaps before Schwann cells and axons (Lewis and Kucenas, 2014). These outcomes implied the essential role of perineurial cells in peripheral nerve regeneration. The participation of other peripheral physical barriers needs to be further investigated. Notably, the molecular investigations of the process of peripheral nerve regeneration suggested that robust inflammation reactions were acutely induced by peripheral nerve injury (Yi et al., 2015, 2017; Xing et al., 2017). Therefore, the distribution of physical barriers following peripheral nerve injury might have close linkage with injury-induced inflammation reactions.

Interestingly, peripheral nerve injury may not only affect physical barriers in the peripheral nervous system, but also induce changes of physical barriers in the central nerve system. It was demonstrated that partial sciatic nerve ligation in rats would induce a remote leakage of the blood-spinal cord barrier possibly by triggering spinal cord inflammatory reactions (Echeverry et al., 2011). These findings connected the pathological changes in peripheral nerves to the entire nervous system and further added the complexity of peripheral nerve injury.

The cellular and molecular alternations of physical barriers after peripheral nerve injury are not well elucidated so far. Correspondingly, there are few attempts to use molecules or chemicals that prevent the disruption of physical barriers as therapeutic treatments of peripheral nerve injury. In contrast, many therapeutic methods have been applied to treat peripheral neuropathies or neurotoxicity by manipulating physical barriers in the peripheral nervous system. For instance, Kashiwamura et al. (2011) treated cultured human peripheral nerve microvascular endothelial cells with glucocorticoids, steroid hormones that could successfully re-establish the blood-brain barrier in inflammatory central nervous system diseases. Glucocorticoids up-regulated the expression of claudin-5, elevated the value of transendothelial electrical resistance, increased the integrity of the blood-nerve barrier, and thus could be used as a potential treatment to blood-nerve barrier disruption-related peripheral neuropathies (Kashiwamura et al., 2011). Shimizu et al. (2014) found that the sera of MMN patients could decrease the amount of claudin-5 protein and the transendothelial electrical resistance in human peripheral nerve microvascular endothelial cells. They further treated human peripheral nerve microvascular endothelial cells with neutralizing antibody against vascular endothelial growth factor (VEGF) prior to the sera of MMN patients and found that treatment with VEGF antibody could up-regulate the expression of claudin-5 and increase the transendothelial electrical resistance (Shimizu et al., 2014). Considering that the disruption of physical barriers is a remarkable characteristic of peripheral nerve injury, it is expected that protective treatments for physical barriers following peripheral nerve injury will benefit the function recovery of injured peripheral nerves. Experiments can be performed to examine whether the direct exposure of injured peripheral nerves to modulators of the blood-nerve barrier will affect the recovery effect of peripheral nerves.

On the other hand, it is worth noting that although physical barriers are critical for maintaining the physiological functions of the nervous system, physical barriers are hampering drug delivery. Therefore, barrier-breaking methods and/or drugs may contribute to the treatment of diseases in the nervous system. For example, tight junction modulating agents, such as sodium caprate, hyperosmolar agent mannitol, zonula occludin toxin, peptidomimetics, and synthetic small interfering RNAs against cellular junctional proteins could temporary break the blood-brain barrier and improve drug delivery (Greene and Campbell, 2016). It was found that a novel synthetic intracellular sigma peptide that binds to and blocks protein tyrosine phosphatases σ could facilitate the delivery of drugs into the spinal cord and benefit the locomotor and urinary functional recovery of injured spinal cord (Lang et al., 2015). Claudin-1-peptidomimetics deriving from the first extracellular loop on claudin-1 could open the perineurial barrier and thus could be used to help the delivery of hydrophilic substances to peripheral nerves (Sauer et al., 2014). Therefore, the alternations of physical barriers in the peripheral nervous system may also have beneficial roles as the transient opening of physical barriers may benefit the penetration of drugs and contribute to the treatment of peripheral neuropathies.

## Author Contributions

All authors listed have made a substantial, direct and intellectual contribution to the work, and approved it for publication.

## Conflict of Interest Statement

The authors declare that the research was conducted in the absence of any commercial or financial relationships that could be construed as a potential conflict of interest.
